# *Pseudomonas* Diversity Within Urban Freshwaters

**DOI:** 10.3389/fmicb.2019.00195

**Published:** 2019-02-15

**Authors:** Mary Batrich, Laura Maskeri, Ryan Schubert, Brian Ho, Melanie Kohout, Malik Abdeljaber, Ahmed Abuhasna, Mutah Kholoki, Penelope Psihogios, Tahir Razzaq, Samrita Sawhney, Salah Siddiqui, Eyad Xoubi, Alexandria Cooper, Thomas Hatzopoulos, Catherine Putonti

**Affiliations:** ^1^Niehoff School of Nursing, Stritch School of Medicine, Loyola University Chicago, Maywood, IL, United States; ^2^Bioinformatics Program, Loyola University Chicago, Chicago, IL, United States; ^3^Department of Biology, Loyola University Chicago, Chicago, IL, United States; ^4^Department of Computer Science, Loyola University Chicago, Chicago, IL, United States; ^5^Department of Microbiology and Immunology, Stritch School of Medicine, Loyola University Chicago, Maywood, IL, United States

**Keywords:** *Pseudomonas*, freshwater, Lake Michigan, heavy metal resistance bacteria, genomics

## Abstract

Freshwater lakes are home to bacterial communities with 1000s of interdependent species. Numerous high-throughput 16S rRNA gene sequence surveys have provided insight into the microbial taxa found within these waters. Prior surveys of Lake Michigan waters have identified bacterial species common to freshwater lakes as well as species likely introduced from the urban environment. We cultured bacterial isolates from samples taken from the Chicago nearshore waters of Lake Michigan in an effort to look more closely at the genetic diversity of species found there within. The most abundant genus detected was *Pseudomonas*, whose presence in freshwaters is often attributed to storm water or runoff. Whole genome sequencing was conducted for 15 Lake Michigan *Pseudomonas* strains, representative of eight species and three isolates that could not be resolved with named species. These genomes were examined specifically for genes encoding functionality which may be advantageous in their urban environment. Antibiotic resistance, amidst other known virulence factors and defense mechanisms, were identified in the genome annotations and verified in the lab. We also tested the Lake Michigan *Pseudomonas* strains for siderophore production and resistance to the heavy metals mercury and copper. As the study presented here shows, a variety of pseudomonads have inhabited the urban coastal waters of Lake Michigan.

## Introduction

Bacteria plays a critical role in the ecosystems of freshwater lakes driving global biogeochemical cycles. As such, numerous surveys of bacterial communities within freshwaters have been conducted, including those examined by Newton et al. (2011) and more recent studies (e.g., Oh et al., 2011; Jones et al., 2012; Han M. et al., 2016; Hayden and Beman, 2016; Lee et al., 2016; Llorens-Marès et al., 2016; Yang et al., 2016; Linz et al., 2017; Ren et al., 2017; Salmaso et al., 2018). Differences in the bacterial communities of these lakes can vary significantly (Newton et al., 2011) as local environmental factors often play a role in structuring these communities (Hayden and Beman, 2016). The community structure of a single lake can vary over time, over the course of a single day (Jones et al., 2012) as well as over years (Linz et al., 2017). Nevertheless, many bacterial groups within freshwaters have a global distribution: *Proteobacteria, Actinobacteria, Bacteroidetes, Cyanobacteria*, and *Verrucomicrobia* (Zwart et al., 1998; Newton et al., 2011). Dominant members of these phyla include the *Proteobacteria* genus *Acinetobacter* (Lee et al., 2016), the acI cluster of *Actinobacteria* (Newton et al., 2007; Neuenschwander et al., 2018), and the *Bacteroidetes* genera *Sphingobacteriales, Sediminibacterium, Fluviicola*, and *Flavobacterium* (Salmaso et al., 2018). These communities, however, can be impacted by disruptions, such as cyanobacterial blooms (Eiler and Bertilsson, 2004; Scherer et al., 2017). Furthermore, bacteria can be introduced into a community via effluent from water treatment plants (e.g., *Escherichia coli* and *Enterococcus*) and stormwater runoff (e.g., *Pseudomonas* and *Sphingomonas*). Such introductions can include species harboring antibiotic resistance genes into the lake ecosystem (Drury et al., 2013; Marti et al., 2013; Fisher et al., 2015; Saarenheimo et al., 2017; Chu et al., 2018; Lorenzo et al., 2018).

The Great Lakes are one of the planet’s largest freshwater ecosystems (Fuller et al., 1995) and as dynamic as coastal oceans (Rao and Schwab, 2007). Not unsurprisingly, microbial surveys of the Great Lakes have found that they share common bacterial diversity with other freshwater habitats (Mueller-Spitz et al., 2009; Mou et al., 2013; Wilhelm et al., 2014; Fisher et al., 2015; Malki et al., 2015a; Newton and McLellan, 2015; Sible et al., 2015). As the Great Lakes are the source of drinking water for millions of North Americans, protection is of critical concern in the face of the many known effects of urbanization on water quality (Foley et al., 2005). This concern is perhaps most evident within Chicago, whose city and surrounding suburbs sprawl along the Lake Michigan shoreline. Lake Michigan waters are routinely monitored (Whitman and Nevers, 2008; Wong et al., 2009; Haack et al., 2013; Oster et al., 2014; Wijesinghe et al., 2015), and the incidence of illness attributed to these waters has been extensively studied (Dorevitch et al., 2011; DeFlorio-Barker et al., 2018). Aside from prompt and thorough testing, contamination via stormwater and fecal pollution has been a consistent concern for all cities along the Lake Michigan shore (Bower et al., 2005; Whitman and Nevers, 2008; Wong et al., 2009; Fisher et al., 2015; Templar et al., 2016). Despite these concerns, the study of Newton and McLellan (2015) found that, at least in the Milwaukee area, the dispersal of bacteria introduced from the urban environment was limited.

Freshwater pseudomonads may be persistent or ephemeral members of the community. Dispersal of pseudomonads from agricultural to non-agricultural environments may be the result of the water cycle (Morris et al., 2008, 2014). As a whole, pseudomonads are often not abundant within freshwater environments (Newton et al., 2011). When pseudomonads are abundant within freshwaters, their source is typically associated with storm waters (Hoadley and McCoy, 1966; Oliveri et al., 1977; Selvakumar and Borst, 2006; Fisher et al., 2015). Culture-independent, 16S rRNA gene amplification surveys of freshwaters, rarely detect pseudomonads indicative of their absence/low abundance. In our prior surveys of the Lake Michigan nearshore waters, *Pseudomonas* was rarely detected via 16S rRNA sequencing (Malki et al., 2015a; Sible et al., 2015). Nevertheless, diverse species of *Pseudomonas* have been isolated from freshwaters (Drucker and Panasyuk, 2006; Morris et al., 2008; Pietsch et al., 2017), including the Great Lakes Erie, Ontario and Superior (Bennett, 1969; Chatterjee et al., 2017). Furthermore, bacteriophage (phage) infectious of *Pseudomonas* species have been routinely found within Lake Michigan nearshore waters (Malki et al., 2015b; Watkins et al., 2018), thus suggesting the presence of the bacterial host. This motivated the study presented here in which *Pseudomonas* species found within Chicago’s nearshore waters of Lake Michigan were sequenced and characterized.

## Materials and Methods

### Sample Collection

Three Chicago public beaches, Loyola Beach, Montrose Beach, and 57^th^ Street Beach, were selected for sampling of the Lake Michigan nearshore waters. These three sites are located 8.9 miles north, 6.1 miles north, and 7.5 miles south of Chicago’s city center, respectively, and include areas for swimming. In addition, Montrose Beach is home to one of Chicago’s boat marinas and a dog beach. Water samples were collected from the swimming areas of the waters at these beaches twice during the summer of 2009 – June 24 and July 14. Water samples were collected near the shore in a depth of 0.5 m using sterile 50 ml centrifuge tubes. Additionally, the Loyola Beach was revisited and sampled on June 3, 2016 after a heavy rain. In addition to collecting samples from the lake (at the same depth sampled in 2009), water samples from two shallow puddles on the beach front were collected. A soil sample was also collected from the beach (a mixed sand and soil beach) by boring a sterile 15 ml centrifuge tube. The soil sample was diluted with 500 mL phosphate buffered saline (PBS) and agitated in a shaking incubator overnight.

### Sample Culture and Strain Isolation

Six different medias were used to plate the water samples and diluted soil sample collected: LB, EMB, Sulfate API, Eugonics (HiMedia Laboratories), Centrimide (HiMedia Laboratories), and Streptococcus (HiMedia Laboratories). For each collection site and date, four plates per media were prepared as follows: 100 μL of water was aliquoted and spread on 1.7% agar plate. Two plates were grown overnight at 25°C and two at 37°C. Seventy-two individual colonies were picked and resuspended in 1 mL of the same media as the plate it was isolated. The resuspended colony was grown overnight in a shaking incubator at the temperature it was previously grown (either 25 or 37°C). Using an inoculation loop, the culture was streaked on an agar plate of the same media from which it was originally isolated and incubated overnight. This process was repeated additional times until a consistent colony morphology was observed. A single colony was selected and grown in liquid culture overnight, shaking, at either 25 or 37°C. This culture was used both for producing a freezer stock [1 mL culture and 1 mL 50/50 (%v/v) glycerol stored at -80°C] and DNA extraction.

### 16S rRNA Gene Sequencing

DNA was extracted from liquid culture using the Mo Bio UltraClean Microbial Kit following the manufacturer’s protocol. DNA concentration was quantified using the Quibit fluorometer. To ascertain the genus and species of the environmental isolate, the 16S rRNA gene sequence was amplified using the 63f and 1387r primers (Marchesi et al., 1998). Amplicons were visualized on a 1.2% agarose gel and purified using the E.Z.N.A.^®^ Cycle-Pure Kit. Sixty-one of the 72 colonies isolated were successfully sequenced; isolates that did not lead to successful DNA extraction and/or PCR were repeated three times before being removed from further investigation. Each amplicon was sequenced by Genewiz (New Brunswick, NJ, United States), using both primers for a 2x coverage; sequences were manually trimmed and paired in Geneious v. 11.0.5 (Biomatters, Ltd., Auckland, New Zealand). The complete amplicon was used to query the 16S ribosomal RNA sequences database via BLASTn. 16S rRNA gene sequences are listed in Supplementary Data Sheet 1.

### *Pseudomonas* Genome Sequencing

Isolates confirmed as belonging to the genus *Pseudomonas* and selected for whole genome sequencing were processed as follows. For each sample, a 1.7% agar LB plate was streaked with a loop (sampling the freezer stock) and grown overnight at 37°C. A single colony was selected from this plate and grown in 1.5 mL of LB overnight, shaking, at 37°C. DNA was extracted using the Qiagen DNeasy UltraClean Microbial Kit following the manufacturer’s protocol. DNA concentration was quantified using the Quibit fluorometer. Sequencing libraries were constructed using the Nextera XT DNA Library preparation kit. The library was sequenced on the Illumina MiSeq platform using the MiSeq Reagent Kit v2 (500-cycles) at Loyola University Chicago’s Genomics Facility (Maywood, IL United States).

### Genome Assembly and Annotation

Raw reads were first trimmed for quality using the tool sickle^1^ and then assembled by SPAdes (v3.10.1) (Bankevich et al., 2012). Contigs less than 500 nucleotides in length were removed from further consideration. Assembled contigs were then manually inspected and individually queried against the nr/nt database via megaBLAST (Supplementary Table 1). Details regarding the number of raw reads, number of contigs, and the assembly N50 scores are listed in Supplementary Table 2. The genome coverage of each assembly was calculated using BBMap’s bbwrap and pileup scripts^2^. Coverage values are also listed in Supplementary Table 2. Each assembly was annotated using the RAST web service with the “Classic RAST” option (Aziz et al., 2008). CRISPR/Cas detection was performed using CRISPRCasFinder using the parameters “general” and “unordered,” which allow a permissive search for CAS genes within contigs respectively (Couvin et al., 2018).

### Prophage Detection and Analysis

Identification of prophage sequences within the genome assemblies was conducted using PHASTER (Arndt et al., 2016). Predicted prophage sequences were queried against the NCBI nr/nt database and compared to each other using progressiveMauve (Darling et al., 2004). Prophage sequences were annotated using the RAST web service with the “Classic RAST” option (Aziz et al., 2008). PCR primers were designed for each of the PHASTER predicted intact prophages using Primer3^3^ and obtained from Eurofins Genomics (Louisville, KY, United States). DNA from each sample was then amplified using the primers to confirm the integration of the predicted prophages.

### Phylogenetic Analyses

All of the Lake Michigan isolate 16S rRNA gene amplicon sequences were aligned using MUSCLE (Edgar, 2004). The 16S rRNA gene, *gyrB, rpoB*, and *rpoD* nucleotide sequences were retrieved from the GenBank annotations for the following strains: *P. aeruginosa* PAO1 (NC_002516), *P. brassicacearum* NFM421 (NC_015379), *P. denitrificans* ATCC 13867 (NC_020829), *P. entomophila* str. L48 (NC_008027), *P. fluorescens* Pf0-1 (NC_007492), *P. fulva* 12-X (NC_015556), *P. mendocina* ymp (NC_009439), *P. poae* RE^∗^1-1-14 (NC_020209), *P. protogens* Pf-5 (NC_004129), *P. putida* KT2440 (NC_002947), *P. savastanoi* pv. phaseolicola (NC_005773), *Pseudomonas* sp. UW4 (NC_019670), *P. stutzeri* A1501 (NC_009434), *P. syringae* pv. tomato str. DC3000 (NC_004578), and *Cellvibrio japonicus* Ueda107 (NC_010995). These gene sequences were concatenated through Geneious and aligned using Clustal Omega (Sievers et al., 2011). All phylogenetic trees were derived using FastTree (Price et al., 2010) through Geneious and visualized in iTOL (Letunic and Bork, 2016).

Average nucleotide identity (ANI) was computed for the 15 sequenced *Pseudomonas* genomes, the aforementioned *Pseudomonas* RefSeq genomes, and the outgroup *C. japonicus* Ueda107 using FastANI (Jain et al., 2018) with the minFrag option set to 30 to accommodate the distance between the *Pseudomonas* strains and the outgroup. The lower triangular matrix generated by the software was then converted to a distance matrix, clustered using UPGMA through the PHYLIP neighbor executable^4^, and visualized in iTOL (Letunic and Bork, 2016). The ANI Calculator (Goris et al., 2007) was also used to confirm results.

### Siderophore Production Assays

To test siderophore production within the Lake Michigan *Pseudomonas* strains, we used the universal siderophore assay with chrome azurol S (CAS) and hexadecyltrimethylammonium bromide (HDTMA) as indicators. Media was prepared as detailed in Louden et al. (2011). In the presence of siderophores, the media will change color from blue to orange. 7 mL of 1.7% agar media was aliquoted into each well of a 6-well plate. Once solidified, 4 μL of the turbid, overnight *Pseudomonas* culture was spotted onto the media and placed in the incubator at 37°C. After ∼48 h, the plates were removed from the incubator and evaluated.

### Heavy Metal Susceptibility Testing

To test the resistance of *Pseudomonas* strains to mercury, the methods of Sone et al. (2013) were employed. Briefly, *Pseudomonas* strains were grown overnight in 30 mL LB, shaking, at 37°C. 200 μL of the overnight culture was suspended in 3 mL LB medium containing HgCl_2_ (Acros Organics; Geel, Belgium) at varied concentrations. Each concentration/strain was cultured in triplicate. Following incubation for 16 h at 37°C, each culture’s absorbance at A_600_ was measured. The same approach was taken for testing the resistance of *Pseudomonas* strains to copper: 200 μL of the overnight culture was suspended in 3 mL LB medium containing CuSO_4_ 5H_2_O (Acros Organics; Geel, Belgium) at varied concentrations. Each concentration/strain was cultured in triplicate for 16 h at 37°C and each culture’s absorbance at A_600_ was measured.

### Antibiotic Susceptibility Testing

The Kirby-Bauer (KB) disk diffusion susceptibility method was used to test the *Pseudomonas* strains’ sensitivity to three antibiotics: Fosfomycin (BD Diagnostics; Sparks, MD, United States), Ciprofloxacin (BD Diagnostics; Sparks, MD, United States), and Vancomycin (BD Diagnostics; Sparks, MD, United States). 200 μL of the bacterial overnight culture was spread on a 1.7% agar LB plate and dried for 5 min. On each plate the three antibiotic disks were placed on the lawn with sterile forceps and incubated overnight at 37°C. Plates were then removed and the zone of inhibition was measured. Susceptibility was determined based upon the BD Sensi-Disk protocol for each chemical. These tests were conducted in duplicate. Three isolates were excluded from the results due to inconsistent growth and subsequent inconclusive test results.

## Results

**FIGURE 1:**
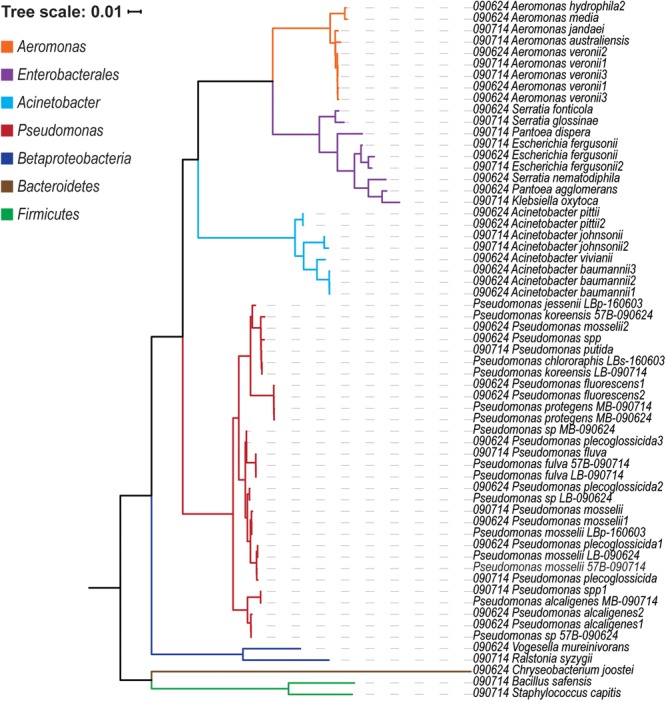
Phylogenetic tree of the 16S rRNA gene sequences from isolates collected.

Water collected from the Chicago nearshore waters of Lake Michigan in the summer of 2009 and 2016 were plated on six different medias. The plated samples were monitored over 5 days in which the number of bacterial colonies was counted. As the medias used selected for different species of culturable bacteria, variation between the growth rates and total growth between media was expected. The “less-selective” medias – LB, Eugonics and EMB – always exhibited more colonization than the “more-selective” Streptococcus, API, and Centrimide medias; this is true across collection days and sampling sites (Supplementary Data Sheet 2). Distinct colonies were selected from the plates and identified by 16S rRNA gene sequencing (Figure 1). The genera identified were representative of species which are known to be present within freshwaters and more critically are amenable to the conditions of culture used in this study. The most abundant genus detected was *Pseudomonas* with several different species identified. Given the number of the pseudomonads recovered, we sequenced the genomes of 15 representatives of the diversity detected, each with a unique 16S rRNA gene sequence. Twelve of the isolates came from samples taken directly from the water, two from puddles on the beach, and one from the soil of the beach. Sequencing statistics, including the number of reads generated, the number of contigs, N50 scores, and coverage can be found in Supplementary Table 2. Table 1 summarizes the 15 genomes sequenced, including their GenBank accession numbers.

**Table 1:** General features of the 15 *Pseudomonas* strains sequenced.

Strain designation	Sample collection details	Genome size (Mbp)	% GC	Proteins	RNAs	WGS project ID
*P. alcaligenes* str. MB-090714	Montrose Beach Jul-14-09	4.01	66.5	3812	60	QJRX00000000
*P. chlororaphis* str. LBs-160603	Loyola Beach Soil Jun-03-16	6.80	62.9	6011	67	QJRW00000000
*P. fulva* str. 57B-090714	57^th^ St Beach Jul-14-09	4.88	61.6	4356	76	QJRV00000000
*P. fulva* str. LB-090714	Loyola Beach Jul-14-09	4.87	61.6	4354	76	QJRU00000000
*P. jessenii* str. LBp-160603	Loyola Beach Jun-03-16	6.77	59.1	5917	72	QJRT00000000
*P. koreensis* str. 57B-090624	57^th^ St Beach Jun-24-09	5.98	60.0	5287	67	QJRS00000000
*P. koreensis* str. LB-090714	Loyola Beach Jul-14-09	6.07	59.9	5328	74	QJRR00000000
*P. mosselii* str. LB-090624	Loyola Beach Jun-24-09	5.77	64.6	5029	74	QJRP00000000
*P. mosselii* str. LBp-160603	Loyola Beach Jun-03-16	5.77	64.2	5105	73	QJRO00000000
*P. protegens* str. MB-090624	Montrose Beach Jun-24-09	6.78	62.2	6057	69	QJRN00000000
*P. protegens* str. MB-090714	Montrose Beach Jul-14-09	6.68	62.4	5975	68	QJRM00000000
*P. soli* str. 57B-090714	57^th^ St Beach Jul-14-09	5.50	64.1	4950	75	QJRQ00000000
*Pseudomonas* sp. 57B-090624	57^th^ St Beach Jun-24-09	7.08	66.4	6342	62	QKRU00000000
*Pseudomonas* sp. LB-090624	Loyola Beach Jun-24-09	5.67	62.1	5028	75	QJRL00000000
*Pseudomonas* sp. MB-090624	Montrose Beach Jun-24-09	6.07	62.0	5417	78	QJRK00000000

*Strain names indicate the beach and sample collection date. The two samples collected from shallow puddles on the beach are designated by “p” and the beach soil by “s.”*

**FIGURE 2:**
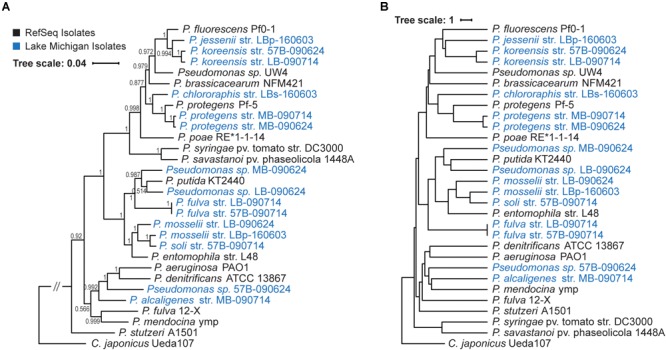
Phylogenetic diversity of 15 Lake Michigan pseudomonads. **(A)** Phylogenetic tree of concatenated 16S rRNA, *gyrB, rpoB*, and *rpoD* gene sequence. Branch support values are shown. **(B)** Hierarchical clustering based upon reciprocal ANI values. RefSeqs are indicated in black font and genomes generated in this study in blue font.

As prior studies have noted that the 16S rRNA gene sequence can be insufficient in distinguishing species within the genus, we employed two methods to determine the species present. First, *Pseudomonas* species can be differentiated based upon the sequence homology of four housekeeping genes: the 16S rRNA gene sequence, *gyrB, rpoB*, and *rpoD* (Gomila et al., 2015). Figure 2A shows the concatenated sequence phylogenetic tree for the 15 genomes sequenced here and complete RefSeq *Pseudomonas* genomes, with *Cellvibrio japonicus* as the outgroup. Second, the ANI was calculated for the 15 genomes sequenced here, RefSeq *Pseudomonas* genomes, and the *C. japonicus* outgroup (Figure 2B). ANI values ranged between 78.5711% (RefSeq genomes *P. savastanoi* pv. phaeseolicola 1448A and *P. aeruginosa* PAO1) and 99.9975% (*P. fulva* str. LB-090714 and *P. fulva* str. 57B-090714). Eight *Pseudomonas* species were identified as well as three isolates for which a species designation could not be made. Two of these species, *Pseudomonas* sp. MB-090624 and *Pseudomonas* sp. LB-090624, clade with *P. putida* (Figure 2A). These two species have a pairwise ANI of 88.3160% and are more similar to each other than any of the other Lake Michigan isolates (Figure 2B). Of the RefSeq sequences included in this analysis, they are most similar to *P. putida* KT2440 (87.8185–90.0128% ANI); this is nevertheless below the 95% threshold expected of strains belonging to the same species. The concatenated sequence phylogenetic tree and ANI analysis, coupled with BLAST results for the individual contigs (Supplementary Table 1), informed the species classifications made. In the case of *P. jessenii* str. LBp-160603, further investigation was required; the gene annotations for this strain were compared to those previously identified for differentiating *P. jessenii* from other close relatives (Verhille et al., 1999) (Supplementary Table 3). Furthermore, upon submission of the 15 genome sequences, NCBI confirmed species designation via ANI against the type strains and proxytype strains that are already in GenBank (Federhen et al., 2016).

The 15 *Pseudomonas* genomes sequenced here vary in size from 4.01 to 7.08 Mbp, encoding 3812 to 6342 protein sequences (Table 1). Genome annotations can be found in Supplementary Table 4 and in GenBank (see Table 1 for accession numbers). No evidence of the CRISPR-Cas system was found. Annotations were compared identifying core subsystems present within the genomes as well as strain specific pathways predicted by the RAST web service (Aziz et al., 2008) (Supplementary Table 5). Comparative analysis revealed 175 subsystems that varied in their presence/absence amongst the 15 strains (Supplementary Table 6). From this list we probed deeper to identify strain- and group-specific attributes distinguishing the Lake Michigan *Pseudomonas* strains. While all genomes contained genes associated with ammonia assimilation, strains varied in the presence of functionality associated with other roles of nitrogen fixation (Supplementary Table 5). Variation between the strains was also observed with respect to sources for sulfur and carbon (Supplementary Table 5). Several of the strains also included gene annotations for the synthesis of achromobactin, aerobactin, and pyoverdine siderophores (Table 2). Evidence for the synthesis of all three of these types of siderophores was found within the genome of *Pseudomonas* sp. MB-090624. In contrast, the genome of *P. mosselii* LBp-160603 does not include any genes associated with siderophore synthesis. Siderophore production was evaluated in the lab using a universal siderophore assay (see section “Materials and Methods”). Siderophore production was prominent within *P. koreensis* str. 57B-090624, *P. fulva* str. LB-090714, *P. fulva* str. 57B-090714, and *P. alcaligenes* str. MB-090714 (Supplementary Data Sheet 3).

**Table 2:** Evidence from strain annotations for the synthesis of siderophores.

	Pa	Pc	Pf57	PfL	Pj	Pk57	PkL	PmL	PmLp	PpM6	PpM7	Ps	P57	PL	PM
Iron siderophore sensor and receptor system		+	+	+	+	+	+	+	+	+	+	+	+	+	+
Siderophore achromobactin		+	+	+	+	+	+					+	+	+	+
Siderophore aerobactin	+		+	+						+	+		+		+
Siderophore pyoverdine		+					+	+		+	+				+

*Pa = *P. alcaligenes* str. MB-090714; Pc = *P. chlororaphis* str. LBs-160603; Pf57 = *P. fulva* str. 57B-090714; PfL = *P. fulva* str. LB-090714; Pj = *P. jessenii* str. LBp-160603; Pk57 = *P. koreensis* str. 57B-090624; PkL = *P. koreensis* str. LB-090714; PmL = *P. mosselii* str. LB-090624; PmLp = *P. mosselii* str. LBp-160603; PpM6 = *P. protegens* str. MB-090624; PpM7 = *P. protegens* str. MB-090714; Ps = *P. soli* str. 57B-090714; P57 = *Pseudomonas* sp. 57B-090624; PL = *Pseudomonas* sp. LB-090624; PM = *Pseudomonas* sp. MB-090624. Presence is indicated by a “+.”*

Genome annotations were further mined for virulence factors and defense mechanisms. All 15 strains encoded for genes associated with bacteriocin (colicin E2 and V) resistance, multidrug efflux pumps, and resistance to fluoroquinolones; resistance to Fosfomycin, Vancomycin, Spectinomycin, and Streptothricin was also detected in several of the strains (Supplementary Table 6). Sensitivity was tested for three of the antibiotics, Ciprofloxacin (a fluoroquinolone), Fosfomycin, and Vancomycin, via the Kirby-Bauer (KB) disk diffusion susceptibility method. As shown in Table 3, all of the strains tested exhibited sensitivity to Ciprofloxacin. However, all of the Lake Michigan *Pseudomonas* genome annotations included gene(s) associated with resistance to fluoroquinolones. While resistance to Fosfomycin was predicted for several strains, resistance was only observed for one of the strains predicted: the *P. protogens* str. MB-090624. Vancomycin resistance was observed for all of the strains tested (Table 3), although this resistance was only identified in three of the strains: *P. jessenii* str. LBp-160603, *P. protogens* str. MB-090624, and *P. protogens* str. MB-090714 (Supplementary Table 6).

**Table 3:** Antibiotic resistance testing.

	Fosfomycin	Ciprofloxacin	Vancomycin	Annotated resistance(s)
*P. alcaligenes* str. MB-090714	S	S	–	C
*P. chlororaphis* str. LBs-160603	S	S	R	F,C
*P. fulva* str. 57B-090714	I	S	–	C
*P. fulva* str. LB-090714	I	S	–	C
*P. jessenii* str. LBp-160603	S	S	R	F,C,V
*P. koreensis* str. 57B-090624	R	S	–	C
*P. mosselii* str. LB-090624	I	S	–	F,C
*P. mosselii* str. LBp-160603	S	S	–	F,C
*P. protegens* str. MB-090624	R	S	–	F,C,V
*Pseudomonas* sp. 57B-090624	S	S	–	C
*Pseudomonas* sp. LB-090624	I	S	R	C
*Pseudomonas* sp. MB-090624	I	S	–	C

*KB test results for Fosfomycin (F), Ciprofloxacin (C), and Vancomycin (V) as sensitive (S), intermediate (I), or resistant (R). (Strains in which no zone of inhibition was detected are indicated by “-.”) Annotated resistances for the three antibiotics tested are listed (from Supplementary Table 6).*

**FIGURE 3:**
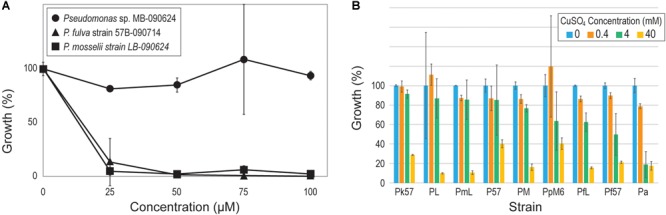
Pseudomonad resistance to **(A)** mercury and **(B)** copper. [Strain abbreviations in **(B)** are those listed in Table 2].

Given prior evidence of co-selection of antibiotic and heavy metal resistance in freshwater bacteria (see review, Di Cesare et al., 2016), we next turned our attention to heavy metal resistance genes within the strains. All 15 strains encoded genes conferring resistance to arsenic, cobalt-zinc-cadmium, and copper. Resistance to chromium was also identified for the *Pseudomonas* strains, with the exception of. the *P. fulva* and *P. mosselii* strains, and the *P. chlororaphis* LBs-160603 strain encoded genes associated with resistance to zinc. Genes associated with resistances to mercury were only annotated for the genome of *Pseudomonas* sp. MB-090624. To test resistance to mercury, this strain as well as one of the *P. fulva* and one of the *P. mosselii* strains were grown in the presence of Hg(II). Even in the presence of 100 μM of mercury, no effect was detected on bacterial fitness within the *Pseudomonas* sp. MB-090624 strain (Figure 3A). Resistance to copper was also tested for nine of the *Pseudomonas* strains, all of which included a coding region annotated for copper tolerance (Figure 3B). Four of the strains, *P. koreensis* str. 57B-090624, *Pseudomonas* sp. LB-090624, *P. mosselii* str. LB-090624, and *Pseudomonas* sp. 57B-090624, saw only minimal reduction in fitness in the presence of high levels of copper (4 mM).

**FIGURE 4:**
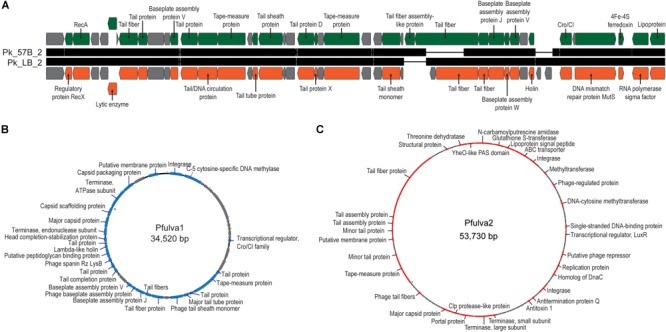
Genome maps of the prophages identified within the Lake Michigan **(A)** *P. koreensis* and **(B,C)** *P. fulva* strains. Hypothetical proteins are indicated in gray.

All 15 of the genome annotations produced by RAST included phage-related genes. To investigate further, each genome was examined using PHASTER (Arndt et al., 2016). Twenty-two intact, 11 incomplete, and one questionable phage sequence (prophage) was identified amongst the 15 genomes. Only *Pseudomonas* sp. LB-090624 did not contain an intact prophage, containing a single incomplete prophage. The prophages identified here exhibited homology to prophages of *Pseudomonas* genomes in GenBank. The one exception being the prophage identified for the *P. alcaligenes* str. MB-090714 sequence; it had only modest sequence homology to an uncultured *Caudovirales* phage (Supplemental Table 7). Focusing on the intact phages identified, 19 were unique. The intact prophage sequences are provided in Supplementary Data Sheet 4. Two of the integrated prophages identified within the *P. koreensis* strains, 35.2 and 37.3 kbp in length, were similar (>95%) to each other and a prophage identified within the sequence of *Pseudomonas moraviensis* BS3668 (GenBank: LT629788) (Figure 4A). Two prophages 34.5 kbp (Figure 4B) and 53.7 kbp (Figure 4C) in length (subsequently referred to as Pfulva1 and Pfulva2, respectively) were identified in both *P. fulva* strains. While their sequences were identical within the two *P. fulva* strains isolated here, they were distinct from sequence within the nr/nt database (Supplementary Table 7). Moreover, no sequence homology was identified between the Pfulva1 and Pfulva2 prophages. The annotated Pfulva1 sequence includes an integrase, C-5 cytosine-specific DNA methylase, and terminase, amongst structural phage genes, lysis proteins (holin and spanin), and hypothetical proteins. Pfulva2 encodes several virulence factors, including a Clp protease, DNA methyltransferase, and antitoxin (Supplementary Tables 8, 9). The presence of these two prophages were further confirmed via PCR amplification.

## Discussion

In 2014 and 2015 we conducted high-throughput 16S rRNA sequencing surveys of the nearshore waters of Lake Michigan, which included the Montrose and 57^th^ Street Beaches, and detected little or no pseudomonads (Malki et al., 2015a; Sible et al., 2015). Thus, *Pseudomonas* is likely not a dominant constituent of these waters. Even so, several different species of *Pseudomonas* have been isolated from other Great Lakes waters via targeted gene sequencing (Chatterjee et al., 2017). Here we have begun a comprehensive catalog of this diversity via whole genome sequencing. Through culture-dependent methods we were able to isolate 11 different *Pseudomonas* species from the Chicago nearshore waters. *P. chlororaphis, P. fulva, P. jessenii, P. koreensis, P. mosselii*, and *P. protegens*, often found in soil, can protect associated plants from fungal and bacterial pathogens (Xie et al., 2003; Deora et al., 2010; Ramette et al., 2011; Liu et al., 2014; Deng et al., 2015; Wu et al., 2018) through the production of diverse molecules (see review, Haas and Défago, 2005). The *Pseudomonas* species isolated here do not commonly cause disease in humans. *P. alcaligenes, P. fulva*, and *P. mosselii*, also commonly found in soil, are rare opportunistic human pathogens (Valenstein et al., 1983; Almuzara et al., 2010; Leneveu-Jenvrin et al., 2013; Suzuki et al., 2013). We did not isolate any strains of the well-studied human pathogen *P. aeruginosa*. This was initially surprising given that *P. aeruginosa* has been detected and/or isolated from stream, river, and lake freshwaters (Pellett et al., 1983; Römling et al., 1994; Pirnay et al., 2005; Khan et al., 2007; Selezska et al., 2012; Magalhães et al., 2016), is prevalent within urban wastewaters (Slekovec et al., 2012), and is the host for phages we previously isolated from these waters (Malki et al., 2015b). However, prior sampling of Lake Erie waters similarly did not find *P. aeruginosa* either (Chatterjee et al., 2017).

Whole genome sequencing of the Lake Michigan *Pseudomonas* isolates provides insight into their evolutionary relationships. As Figure 2 shows, these strains represent a diversity of species within the *Pseudomonas* tree. As prior studies have found that 16S rRNA sequences alone cannot distinguish between *Pseudomonas* species (Mulet et al., 2012; Gomila et al., 2015), two methods were employed here: the concatenated housekeeping genes and whole genome ANI. Only the two strains identified as *P. protegens*, the two strains identified as *P. koreensis*, and the two strains identified as *P. fulva* have a pairwise ANI value exceeding the threshold demarking species delineation (Richter and Rosselló-Móra, 2009). While phylogenetic and ANI analyses both identified the *P. fulva* strains here within the *P. putida* group, they do not clade with the RefSeq *P. fulva* 12-X strain. Prior studies evaluating the ANI (e.g., Han N. et al., 2016; Peña et al., 2016) and phylogeny for this genus (Gomila et al., 2015) have likewise found the *P. fulva* 12-X genome distant from the *P. putida* group. Until quite recently, this was the only complete genome for the species available. Earlier this year, another complete genome was produced: *P. fulva* FDAARGOS_167 (NZ_CP014025), isolated from sputum of a Cystic Fibrosis patient (per the genome’s GenBank record metadata). We found that this new *P. fulva* strain also has an ANI value less than that expected for two representatives of the same species (Supplementary Data Sheet 5), suggesting that *P. fulva* 12-X is a distinct species (i.e., not *fulva*). The three isolates for which a strain determination could not be made – *Pseudomonas* sp. MB-090624, *Pseudomonas* sp. LB-090624, and *Pseudomonas* sp. 57B-090624 – may be representatives of new species.

Investigation of the annotations generated for the 15 Lake Michigan genomes identified unique features including metabolic and nutrient utilization pathways (Supplementary Table 6). Prevalent amongst the Lake Michigan *Pseudomonas* strains is the presence of gene annotations for the synthesis of siderophores (Table 2). We were able to verify siderophore production within four of the 14 strains containing annotations for siderophores using the universal siderophore assay (Supplementary Data Sheet 3). It is important to note that siderophore production within the other 10 strains is likely; however, we were unable to cultivate these strains on the universal siderophore assay media. Although these strains were cultivated routinely under the same laboratory conditions, the universal siderophore media may exclude a vital nutrient/resource for growth. Moreover, it is well-known that species-specific siderophore systems exist amongst the pseudomonads (Meyer et al., 2002). Siderophore production within most of the pseudomonads from Lake Michigan suggests a possible role in enhancing iron abundance within the water column, similar to speculations for siderophore-producing bacteria within marine surface waters (see review, Ahmed and Holmström, 2014). Furthermore, recent evidence found that siderophores play a role in the interactions between co-occurring *Pseudomonas* strains and likely drive diversification in nature (Butaitė et al., 2017).

The Lake Michigan pseudomonads encode for several antibiotic resistance genes (Supplementary Table 6). Fluoroquinolone resistance was identified within the annotations of all 15 Lake Michigan *Pseudomonas* strains. Prior studies have noted its prevalence amongst clinical *Pseudomonas* samples (Horii et al., 2005; López-Causapé et al., 2018) as well as aquatic isolates (Adachi et al., 2013; Johnning et al., 2015; Chu et al., 2018). KB tests performed here using the fluoroquinolone Ciprofloxacin, however, revealed that all of the strains were actually sensitive to the antibiotic (Table 3). The annotated fluoroquinolone resistance clearly does not confer resistance to Ciprofloxacin. The Lake Michigan pseudomonads may be resistant to other fluoroquinolones not tested here, as a prior study found *P. aeruginosa* strains vary in their sensitivity to the fluoroquinolones Ciprofloxacin, Levofloxacin, and Moxifloxacin (Grillon et al., 2016). While the genome annotations also indicated that some strains encode for resistance to Fosfomycin and Vancomycin, KB diffusion assays were often found to contradict these predictions (Table 3). KB assays found that all 15 of the strains are resistant to Vancomycin, nine exhibiting no inhibition of growth. Therefore, the annotations alone cannot definitively characterize a strain’s sensitivities to antibiotics. Correlating mutations within these coding regions and confirmed sensitive and resistant strains is necessary to improve annotations and antibiotic resistance predictions.

As previous research has found, heavy metal resistance genes can cause positive selection of antibiotic resistance genes (Baker-Austin et al., 2006; Pal et al., 2015; see review Di Cesare et al., 2016). The Lake Michigan *Pseudomonas* strains encode for resistance of several heavy metals. In the bioinformatic study of Pal et al. (2015) investigating co-occurrence patterns of resistance genes, the only metal resistance genes commonly co-occurring with antibiotic resistance genes were for mercury resistance. Only one of the Lake Michigan *Pseudomonas* strains, *Pseudomonas* sp. MB-090624, was shown to tolerant of high levels of mercury (Figure 3A). Mercury emissions remain a concern in the Chicago area (Sullivan and Mason, 1998; Gratz et al., 2013); mercury emissions within the area did not see strong regulation until 2006 with the Illinois EPA mercury and Clean Air Interstate Rule. Recently, an increased level of mercury in Lake Michigan fish has been observed (Blukacz-Richards et al., 2017). The multiple heavy metal resistance genes identified within the pseudomonads may prove beneficial within these urban waters in response to potential contaminations.

As our group’s prior work in these waters has focused on isolation and characterization of phages, and in particular *Pseudomonas* infecting phages (Malki et al., 2015b; Watkins et al., 2016, 2018), we were not surprised to find prophages within the 15 Lake Michigan *Pseudomonas* genomes. The *P. koreensis* prophage, identified in isolates from sampling sites 17 mi apart on two separate collection dates, may be (or have been at that time) a frequent member of the Lake Michigan nearshore community. We may hypothesize this to be true for the Pfulva1 and Pfulva2 prophages as well (Figure 4B,C). The *P. fulva* prophages most closely resemble *Pseudomonas* phage φCTX and the unclassified Cvm10 virus *Enterobacter* phage Arya, respectively. It is important to note that these similarities are tenuous; only ∼50% of these prophages exhibit sequence homology to a GenBank record. Novel prophages with < 50% sequence homology to any annotated phage or prophage sequence were also identified within the *P. alcaligenes* str. MB-090714, *P. koreensis* str. 57B-090624, *P. protogens* str. MB-090624, *Pseudomonas* sp. 57B-090624, and *Pseudomonas* sp. MB-090624 genomes (Supplementary Table 7). This hints at a large reservoir of phages within freshwaters that have yet to be discovered. Recent efforts have begun to isolate and characterize freshwater phages, in particular *Pseudomonas* infecting phages (e.g., Mäntynen et al., 2015; Lu et al., 2017).

*Pseudomonas* is a cosmopolitan genus, and while not commonly abundant within freshwater environments (Newton et al., 2011), it has been isolated from numerous freshwater lakes including the Great Lakes (Bennett, 1969; Chatterjee et al., 2017). Here we have presented the first whole genome sequences of pseudomonads from the Great Lakes. Given the anthropogenic impact on the Chicago nearshore waters, it is not possible to determine if these isolates are constituents of the microbial community or ephemeral members recently introduced. Nevertheless, pseudomonads’ ability to breakdown and utilize a wide variety of organic compounds serves them well, especially in varied conditions such as Chicago winters when the nearshore waters often freeze. As the study presented here shows, a variety of pseudomonads have inhabited the coastal waters of Lake Michigan.

## Author Contributions

MB extracted and prepared isolates for sequencing. RS, BH, MA, AA, MKh, PP, TR, SSi, SSa, EX, TH, and CP performed genome assemblies and annotations. MKo and AC collected samples. MB and LM performed phenotype assays. MB, LM, and CP conducted genome sequence analyses and drafted the manuscript. All authors contributed to the editing of the manuscript and approve of the final manuscript.

## Conflict of Interest Statement

The authors declare that the research was conducted in the absence of any commercial or financial relationships that could be construed as a potential conflict of interest.

## Footnotes

1 https://github.com/najoshi/sickle

2 https://sourceforge.net/projects/bbmap/

3 http://bioinfo.ut.ee/primer3-0.4.0/

4 http://evolution.genetics.washington.edu/phylip.html
