# Supplementary material for: Pseudomonas Diversity Within Urban Freshwaters
Source: Front Microbiol. 2019 Feb 15;10:195. doi: 10.3389/fmicb.2019.00195 (PMC6384249; doi:10.3389/fmicb.2019.00195)
Supplement: Supplementary file 2 [file Table_2.DOCX]

| **Species** | **SRA Accession #** | **# read pairs** | **WGS Accession #** | **Genome Size (Mbp)** | **# contigs** | **N50** | **Coverage** |
| --- | --- | --- | --- | --- | --- | --- | --- |
| *P. alcaligenes* strain MB-090714 | SRR7250122 | 834608 | QJRX00000000 | 4.01 | 16 | 443490 | 92.65 |
| *P. chlororaphis* strain LBs-160603 | SRR7250121 | 754953 | QJRW00000000 | 6.80 | 25 | 644656 | 50.67 |
| *P. fulva* strain 57B-090714 | SRR7250119 | 801579 | QJRV00000000 | 4.88 | 47 | 195517 | 69.02 |
| *P. fulva* strain LB-090714 | SRR7250120 | 1068525 | QJRU00000000 | 4.87 | 40 | 192201 | 93.01 |
| *P. jessenii* strain LBp-160603 | SRR7250118 | 891277 | QJRT00000000 | 6.77 | 49 | 327702 | 60.44 |
| *P. koreensis* strain 57B-090624 | SRR7250117 | 882166 | QJRS00000000 | 5.98 | 38 | 339549 | 67.31 |
| *P. koreensis* strain LB-090714 | SRR7250116 | 800821 | QJRR00000000 | 6.07 | 47 | 300178 | 59.66 |
| *P. mosselii* strain LB-090624 | SRR7250114 | 612265 | QJRP00000000 | 5.77 | 82 | 156666 | 45.94 |
| *P. mosselii* strain LBp-160603 | SRR7250113 | 894472 | QJRO00000000 | 5.77 | 52 | 257006 | 67.14 |
| *P. protegens* strain MB-090624 | SRR7250110 | 634320 | QJRN00000000 | 6.78 | 44 | 259492 | 42.80 |
| *P. protegens* strain MB-090714 | SRR7250109 | 911328 | QJRM00000000 | 6.68 | 36 | 545477 | 61.03 |
| *P. soli* strain 57B-090714 | SRR7250115 | 645027 | QJRQ00000000 | 5.50 | 57 | 247468 | 50.60 |
| *Pseudomonas* sp. 57B-090624 | SRR7250112 | 651078 | QKRU00000000 | 7.08 | 189 | 73258 | 38.85 |
| *Pseudomonas* sp. LB-090624 | SRR7250111 | 787991 | QJRL00000000 | 5.67 | 87 | 157906 | 60.29 |
| *Pseudomonas* sp. MB-090624 | SRR7250108 | 936889 | QJRK00000000 | 6.07 | 119 | 95291 | 68.35 |

**Supplementary Data Sheet 3.** Genome statistics for the 15 Lake Michigan *Pseudomonas* strains sequenced in this study.
