# Supplementary material for: Pseudomonas Diversity Within Urban Freshwaters
Source: Front Microbiol. 2019 Feb 15;10:195. doi: 10.3389/fmicb.2019.00195 (PMC6384249; doi:10.3389/fmicb.2019.00195)
Supplement: Supplementary file 3 [file Table_3.DOCX]

| **Feature** | ***P. koreensis***^1,2^ | ***P. moraviensis***^2^ | ***P. jessenii***^1,2,3^ | ***P. donghuensis***^4^ | ***P. fluorescens***^3,5,6^ | ***P. jessenii* LBp-160603** |
| --- | --- | --- | --- | --- | --- | --- |
| Oxidase | + | + | + | + | + | + |
| Nitrate Reductase | - | - | + | + | + | + |
| Gelatin Hydrolysis | d | + | - | + | + | - |
| Arginine dihydrolase | + | - | + | ND | - | + |
| Lecithinase | d | - | - | ND | +/d | - |
| Pyocyanin | ND | ND | - | ND | - | - |
| Median GC % | 59.8 | 60.1 | 59.7 | 62.4 | 60.3 | 59.06 |
| Median length (Mb) | 6.3 | 6.1 | 6.5 | 5.6 | 6.3 | 6.8 |

**Supplemental Data Sheet 4.** Differentiating features for the *P. jessenii* species and related species. Here “+” indicates minimum 90% of strains positive for the trait and “-“ indicates less than 10% positive for trait. “d” indicates that 11-89% of strains are positive for the trait and “ND” indicates no data was found for the given trait. *Note, aside from positive oxidase, nitrate and arginine tests *P. fluorescens* shows a high degree of contradiction among strains for remaining features. Literature referenced: [1] Soon, W.K., Kim, J.S., Park, I.C., Yoon, S.H., Park, D.H., Lim, C.K., et al. (2003) *Pseudomonas koreensis* sp. nov., *Pseudomonas umsongensis* sp. nov. and *Pseudomonas jinjuensis* sp. nov., novel species from farm soils in Korea. Int. J. Syst. Evol. Microbiol. 53, 21-27. [2] Tvrzova, L., Schumann, P., Sproer, C., Sedlacek, I., Pacova, Z., Sedo, O., et al. (2006) *Pseudomonas moraviensis* sp. nov. and *Pseudomonas vranovensis* sp. nov., soil bacteria isolated on nitroaromatic compounds, and emended description of *Pseudomonas asplenii*. Int. J. Syst. Evol. Microbiol. 56, 2657-2663. [3] Verhille, S., Baida, N., Izard, D., and Leclerc, H. (1999) Taxonomic Study of Bacteria Isolated from Natural Mineral Waters: Proposal of *Pseudomonas jessenii* sp. nov. and *Pseudomonas mandelii* sp. nov. System. Appl. Microbiol. 22, 45-58. [4] Gao, J., Xie, G., Peng, F., and Xie, Z. (2015) *Pseudomonas donghuensis* sp. nov., exhibiting high-yields of siderophore. Antonie Van Leeuwenhoek 107, 83-94. [5] Bossis, E., Lemanceau, P., Latour, X., and Gardan, L. (2000) The taxonomy of *Pseudomonas fluorescens* and *Pseudomonas putida*: current status and need for revision. Agronomie 20, 51-63. [6] Soesanto, L., Mugiastuti, E., and Rahayuniati, R.F. (2011) Biochemical Characteristics of *Pseudomonas fluorescnes* P60. J. Biotec. Biodivers. 2, 19-26.
