# Supplementary figures and images for: Pseudomonas Diversity Within Urban Freshwaters
Source: Front Microbiol. 2019 Feb 15;10:195. doi: 10.3389/fmicb.2019.00195 (PMC6384249; doi:10.3389/fmicb.2019.00195)

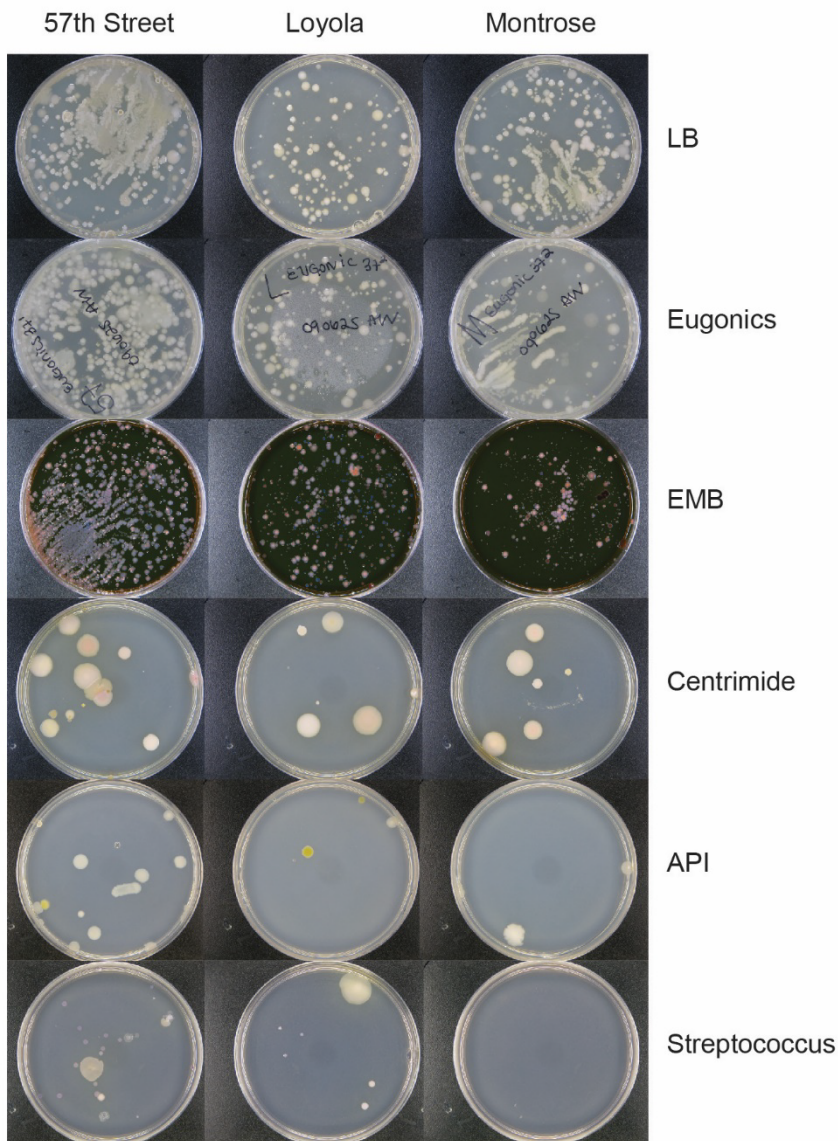

**Supplementary Image 1.** Colonies grown on selective medias for the three beaches sampled on June 24, 2009.

Supplement: Supplementary file 10 [file Data_Sheet_2.PDF]
