# Supplementary material for: Pseudomonas Diversity Within Urban Freshwaters
Source: Front Microbiol. 2019 Feb 15;10:195. doi: 10.3389/fmicb.2019.00195 (PMC6384249; doi:10.3389/fmicb.2019.00195)
Supplement: Supplementary file 11 [file Data_Sheet_3.PDF]

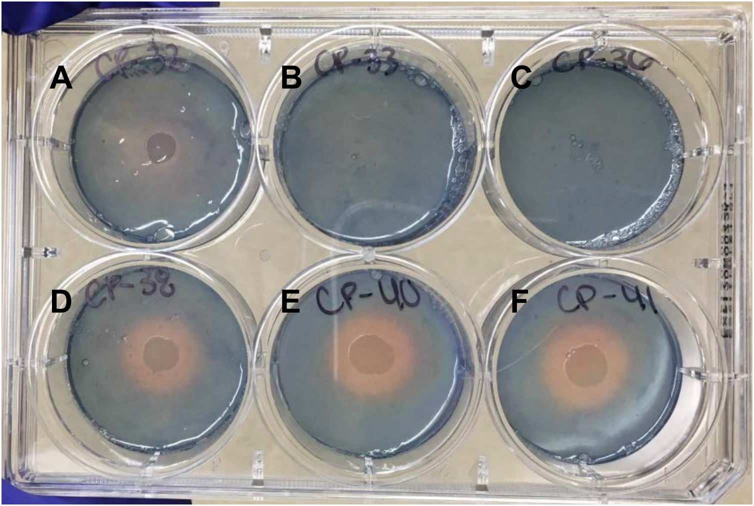


**Supplementary Image 2.** Siderophore production was evaluated using a universal siderophore assay (Louden et al. 2011) for Lake Michigan *Pseudomonas* strains: **(A)** *P. alcaligenes* str. MB-090714, **(B)** *Pseudomonas* sp. 57B-090624, **(C)** *P. protogens* str. MB-090624, **(D)** *P. koreensis* str. 57B-090624, **(E)** *P. fulva* str. LB-090714, and **(F)** *P. fulva* str. 57B-090714.
