# Supplementary material for: Pseudomonas Diversity Within Urban Freshwaters
Source: Front Microbiol. 2019 Feb 15;10:195. doi: 10.3389/fmicb.2019.00195 (PMC6384249; doi:10.3389/fmicb.2019.00195)
Supplement: Supplementary file 13 [file Data_Sheet_5.PDF]

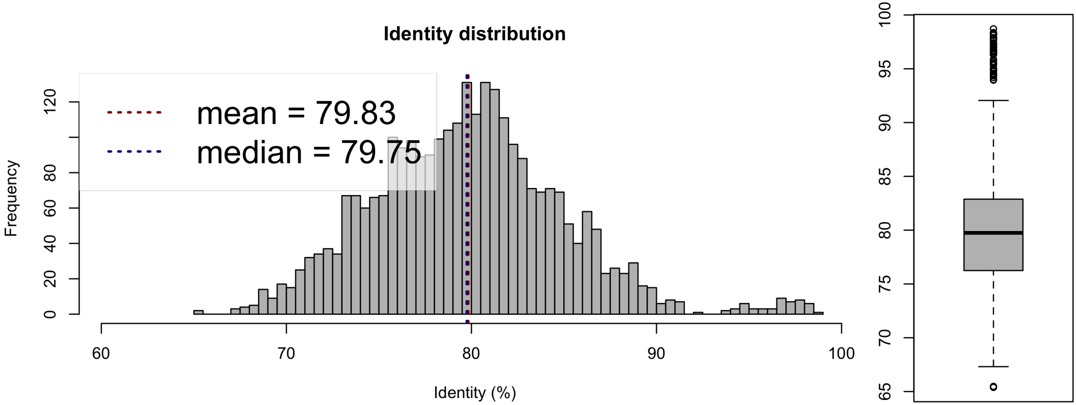

Supplementary Image 3. ANI computed by for the genome sequences of *P. fulva* 12-X (NC_015556) and *P. fulva* FDAARGOS_167 (NZ_CP014025). Calculations performed using the ANI calculator (Goris *et al*. 2007).
